# Matters of the Mind: A Look Into the Life of Sigmund Freud

**DOI:** 10.7759/cureus.71562

**Published:** 2024-10-15

**Authors:** Laresh N Mistry, Shreyas Neelkanthan, Shantanu S Deshpande, Ashwin M Jawdekar, Pakhi P Shah, Nikita A Khachane

**Affiliations:** 1 Department of Pediatric and Preventive Dentistry, Bharati Vidyapeeth (Deemed to be University) Dental College and Hospital, Navi Mumbai, IND

**Keywords:** child, freudian theory, pediatric dentistry, psychoanalysis, psychological theory, psychology

## Abstract

This biography of Sigmund Freud examines the life and contributions of the individual recognized as the progenitor of psychoanalysis, analyzing his significant influence on the fields of psychology, culture, and the comprehension of the human psyche. Sigmund Freud was born in 1856 in Freiburg, Moravia. His early education and burgeoning interest in the field of medicine established a critical foundation for the development of his innovative theories. Following his academic pursuits with distinguished individuals such as Jean-Martin Charcot in Paris, Freud formulated his theories regarding the unconscious mind, mechanisms of repression, and the importance of dreams. Freud's theoretical framework presented constructs such as the id, ego, and superego, fundamentally transforming our understanding of human behavior and motivation. The implementation of innovative methodologies, including free association and transference, has significantly contributed to the evolution of contemporary psychotherapy, facilitating the exploration of inner conflicts and unconscious desires among individuals. In the face of criticism and controversy, Freud's theories exerted a significant influence on psychology, as well as on art, literature, and popular culture, thereby shaping the intellectual landscape of the 20th century. The article further investigates Freud's personal life, encompassing his familial relationships. This also elucidates the diverse benchmarks of human psychiatry that were established during the era of Sigmund Freud. This review characterizes Freud as a multifaceted individual, a trailblazer whose contributions to the understanding of the human psyche persist in relevance, inciting discussions and encouraging further investigation across diverse disciplines. The narrative provides a comprehensive examination of Freud's life, contributions, and lasting impact, emphasizing his significance as a pivotal intellectual whose ideas continue to shape modern discourse surrounding identity, sexuality, and mental health.

## Introduction and background

Psychology is the scientific study of the mind and behavior, aiming to understand how people think, feel, and behave. In pediatric dentistry, applying psychological principles is essential for managing children's behavior, alleviating anxiety, and fostering a positive outlook toward dental care [1].

Studying child psychology is essential because childhood is a critical time for brain development, emotional growth, and learning. Children's minds are still growing, and what happens during their early years can shape their future mental and physical health [2]. By understanding how children think and behave, doctors and other healthcare professionals can diagnose developmental problems early, help kids manage emotional issues, and ensure that they grow up healthy and well-adjusted [3].

In pediatric dentistry, understanding child psychology is crucial because treating children is different from treating adults [3]. Children often feel scared or anxious when going to the dentist, and they may not know how to express their fears or discomfort. By studying child psychology, pediatric dentists learn how to communicate with children in ways that make them feel safe and comfortable [2]. This helps the dentist build trust with the child, reduce their anxiety, and make the dental visit a positive experience. Also, understanding child development allows pediatric dentists to know how a child's oral habits (such as thumb-sucking or improper brushing) may affect their future dental health [2].

For example, if a child struggles with attention, learning, or social skills, understanding child psychology can help identify conditions such as attention deficit hyperactivity disorder (ADHD) or autism early on. Early intervention can then improve outcomes, which is why child psychology is an important part of medical science. A pioneer in understanding the intricacies of child psychology was the valuable input by Dr. Sigmund Freud. His concepts of understanding the mindset of a child opened new horizons for the psychologist, and the behaviors of children could be reasoned with co-relating their age and level of psychological development. He was one of the most influential figures of the 19th and 20th centuries. His work has left a lasting imprint on psychology, psychiatry, philosophy, and even popular culture [4]. Next, we will explore a detailed account of Freud's life, his educational journey, and his major contributions to the fields of psychology and psychoanalysis.

Sigismund Schlomo Freud (1856-1939), later known as Sigmund Freud, was born on May 6, 1856, in Freiberg, Moravia (now the Czech Republic), to Jewish parents. His family moved to Vienna when he was four years old. From an early age, Freud showed intellectual promise, particularly in the study of languages and medicine. He attended the University of Vienna, where he pursued a medical degree, focusing on neurology [5].

In 1873, at the age of 17, Freud entered the University of Vienna, one of Europe's leading centers for scientific research. Initially, Freud had intended to study law, but his interest soon shifted to medicine and biology under the influence of his mentors. He was particularly inspired by the scientific and empirical methods that were becoming prominent in European universities at the time [4].

In 1881, after a protracted academic journey that took him longer than anticipated due to his involvement in research, Freud earned his medical degree. Following his graduation, he began working at the Vienna General Hospital, focusing primarily on neurology [5].

In 1885, Freud received a scholarship to study under the renowned French neurologist Jean-Martin Charcot at the Salpetriere Hospital in Paris. Charcot was a leading expert on hysteria and hypnosis, and his work had a profound influence on Freud's thinking about the nature of mental disorders [5].

Upon his return to Vienna in 1886, Freud opened a private practice specializing in neurology and the treatment of hysterical and neurotic patients [5]. It was during this period that Freud began collaborating with another Viennese physician, Josef Breuer, on the treatment of hysteria. Their joint work, particularly with a famous case known as "Anna O," led to the development of what would become the foundation of psychoanalysis [6].

During his time in medical school, Freud worked closely with influential figures such as Ernst Brücke and Jean-Martin Charcot [5]. Charcot, a French neurologist, introduced Freud to the concept of hysteria and hypnosis, laying the groundwork for his later interest in the unconscious mind [7].

Freud theorized that early human societies were structured around a moral code centered on a totemic figure [8].

This totem emerged from feelings of guilt and remorse after the symbolic killing of the community's founder, often depicted as a father figure. The act of foundational parricide gave rise to emotions that shaped social connections and collective moral principles. Over time, this event was reinterpreted and transformed in various ways, impacting the evolution of social identities throughout history [8].

When examining the moral foundations of postcolonial African societies, it becomes evident that they are shaped by a combination of precolonial customs, the impact of European colonial rule, and the counterforces of the independence movements [9].

Freud's model centers on collective guilt as key to the formation of society, but the authors of this article propose that in the context of 20th-century African societies, the notion of victimization, rooted in the historical impact of colonialism and its aftermath, plays a pivotal role in shaping social identities and moral frameworks [8].

This transition from guilt to victimhood mirrors the distinct historical experiences of these societies [10]. To explore Freud's ideas on societal development further, his work Totem and Taboo is a key resource [8].

Sigmund Freud is widely considered the founder of psychoanalysis, a field within psychology focused on treating mental disorders by examining unconscious aspects of the mind that individuals are typically unaware of [4]. Freud's foundational concepts in psychoanalysis have deeply influenced our culture and understanding since they were first introduced in the medical field [11].

The terms unconscious, id, ego, superego, Oedipus complex, sexual urges, death wishes, repression of emotions, transference, defense mechanisms, and dream interpretation are widely recognized and accepted by many mental health professionals [12]. Nevertheless, psychoanalytic theories offer valuable insights for analyzing literary works for several reasons [13].

Firstly, psychoanalytic theory allows for connections between the underlying, often unconscious content of a novel or poem and the text's overt, conscious elements [14]. Secondly, it links the repression of emotions and the unveiling of unconscious factors either to the characters within a work or to the authors themselves [15]. Thirdly, theories of sexuality can be related to representations of emotional distress and mental dysfunctions, including psychological complaints or manifestations of madness [16]. Fourthly, the Oedipus complex significantly impacts gender dynamics and relationships, such as those portrayed in Shakespeare's Hamlet [14]. Lastly, there is a belief that analyzing psychological components is more crucial for critically reading literary works than other social or historical perspectives [13].

Sigmund Freud famously criticized religious beliefs across various faiths as manifestations of wish fulfillment [17].

Religious beliefs are driven by the desire for a heavenly father figure who provides care and protection, similar to how an earthly father safeguards and disciplines us to ensure our well-being [18].

Freud's approach to critiquing religion often resembled a gunman firing indiscriminately, hoping that some of his arguments would land despite acknowledging that many might miss the mark [17]. He was firmly convinced that religion was an illusion that ultimately caused more harm than good [19].

Freud's theory of religious belief should be assessed in its entirety rather than as isolated claims. It is widely accepted that Freud was an atheist who denied the existence of a deity and was intrigued by the question of why people hold religious beliefs [17]. Unlike beliefs in celestial bodies such as the moon or Mars, which do not present a similar puzzle, Freud argued that the existence of a god is a construct of human desire [18]. He proposed a naturalistic explanation for this phenomenon, with wish fulfillment being a coherent component of his broader theory [14].

This view is reinforced by the fact that religious beliefs often involve a powerful father figure who can manage both the positive and negative aspects of our lives, something that aligns with deep-seated human desires [17,18]. This does not imply that religion lacks significance or that spirituality is not an inherent aspect of human existence [20]. The concept of a personal father figure in Western religions is significantly challenged by Freudian critiques [17].

The psychoanalytical theory was developed by Freud between the 19th and 20th centuries. While treating patients with conditions such as hysteria and neurosis, Freud noticed that much of their behaviors and emotions seemed to be affected by thoughts and feelings. He called this the "unconscious mind" [15]. Freud believed that many of our actions are driven by hidden desires, especially those linked to things such as sex and aggression [20]. Through his work with patients, Freud used methods such as free association (letting people speak freely), interpreting dreams, and analyzing slips of the tongue (now known as "Freudian slips") [21].

He introduced the idea of repression, where painful memories or uncomfortable desires are pushed out of our conscious mind. This concept became a key part of his psychoanalytic theory [15].

## Review

Contributions Sigmund Freud made to psychoanalysis

The Unconscious Mind

Freud believed that a significant part of our thoughts, desires, and memories exist in the unconscious, meaning we are not fully aware of them, yet they influence our behavior [15].

Dream Interpretation

Freud saw dreams as a "royal road" to the unconscious, thinking they reveal hidden desires or unresolved conflicts. He developed a method to analyze dreams to better understand the unconscious mind [14].

Repression

Freud introduced the idea of repression, where painful or troubling memories and desires are pushed out of conscious awareness. He believed this process could lead to psychological problems if the repressed material was not properly addressed [15].

Free Association

Freud developed the technique of free association, where patients are encouraged to speak freely about their thoughts without filtering or judging them. This process helps uncover hidden thoughts and unresolved emotions [8].

The Oedipus Complex

Freud proposed that boys, during early childhood, develop unconscious feelings of desire for their mother and jealousy toward their father, which he called the Oedipus complex. He believed that this was a key stage in personality development [16].

Psychosexual Development

Freud suggested that human development occurs in stages focused on different bodily areas, such as the mouth, anus, and genitals. He believed that unresolved conflicts during any of these stages could lead to personality issues in adulthood [16].

Freud's theories provided a foundation for the study of psychoanalysis, although some ideas have been debated or modified by modern psychologists.

Sigmund Freud's structural model of the psyche that divides the mind into three parts: the id, the ego, and the superego

The Id

According to Freud, the id is the most primitive part of our mind. It represents our basic instincts, drives, and desires, such as hunger, aggression, and the need for pleasure.

The id operates entirely unconsciously and seeks immediate gratification without considering consequences or morals [20].

The Ego

The ego develops as we grow and learn to deal with reality. It works to balance the desires of the id with the rules of the real world. The ego helps us make rational decisions by considering the long-term effects of our actions. It operates in both the conscious and unconscious parts of the mind [20].

The Superego

The superego is like our internal moral compass. It develops as we absorb the values and rules of society, typically taught by parents and authority figures. The superego judges our actions and thoughts, creating feelings of guilt or pride. It pushes us to act according to moral standards, sometimes in conflict with the id's desires [20].

Freud suggested that the id, ego, and superego constantly interact. The id demands instant satisfaction, the superego reminds us of moral standards, and the ego tries to find a practical balance. This constant conflict between the three can lead to anxiety, and the ego uses defense mechanisms to cope with it [22]. Sigmund Freud wrote several influential books that contributed to the shaping of modern psychology and the understanding of the human mind. His work explored key concepts such as the unconscious mind, dreams, and personality development.

Some of his most important books, including when they were written, what they were about, and why they are significant

The Interpretation of Dreams (1899)

In this book, he said that dreams are a window into the unconscious mind. He also argued that dreams do have some hidden desires, fears, and unresolved conflicts. Freud believed that by understanding dreams, we can understand better the unconscious forces that shape behavior. This book was significant because it introduced one of Freud's most famous concepts, the unconscious mind. It laid the foundation for his later work in psychoanalysis and was the first major publication to outline his theory of dream analysis [14].

Three Essays on the Theory of Sexuality (1905)

In this book, he explored human sexual development, presenting Freud's theory that children pass through different stages focused on various parts of the body that were described as oral, anal, and phallic stages of psychosexual development. He introduced concepts such as the Oedipus complex, where boys experience desires for their mothers, and the Electra complex, where girls experience desires for their fathers. It was very much controversial because it challenged Victorian views on childhood and sexuality. Freud's ideas about psychosexual stages became central to psychoanalysis and subsequently influenced how psychologists understood human development [16].

Totem and Taboo (1913)

In this book, Freud applied psychoanalytic theory, exploring the connection between ancient cultures and modern psychology. He analyzed totems (sacred symbols) and taboos (forbidden behaviors) in tribal societies, which suggests that primitive behaviors provide human psychological development. This book was important because it showed how Freud's theories could be applied beyond individual psychology to explain cultural and social phenomena. It also stated his belief in unconscious desires, especially related to sex and aggression [8].

The Ego and the Id (1923)

This book introduced Freud's structural model of the mind, dividing it into three parts: the id, the ego, and the superego. The id represents basic instincts, the ego deals with reality, and the superego reflects our moral standards. He used this model to explain how these different parts of the psyche interact and influence behavior. This work was crucial because it provided a clear framework for understanding the human mind's structure. It helped explain the constant conflict between our desires (id), morals (superego), and practical decision-making (ego) [20].

Civilization and Its Discontents (1930)

In this book, Freud explored the tension between individual desires and the demands of society. He argued that civilization requires people to suppress their instinctual drives, especially those related to aggression and sexuality, which leads to feelings of unhappiness or discontent. This book is important because it addresses the broader social implications of Freud's theories. It highlights the idea that societal rules and norms often conflict with our natural desires, creating psychological distress [18].

Freud's books laid the foundation for modern psychoanalysis and profoundly influenced psychology and psychiatry. His exploration of the unconscious mind, human development, and the conflict between personal desires and societal rules continues to resonate in the field of mental health today.

Sigmund Freud's theories, although developed over a century ago, continue to influence modern psychology and therapy. However, many of his ideas have been updated, adapted, or even criticized by contemporary researchers and therapists.

The Unconscious Mind

Freud's idea of the unconscious mind remains a central concept in modern psychology. However, today's view of the unconscious includes not only repressed desires but also automatic processes that influence our behavior, such as habits or biases we are not aware of [23].

Dream Interpretation

Freud believed dreams reveal hidden parts of our unconscious mind. Today, while dream interpretation is less central in mainstream psychology, some therapists still use it as a tool in psychoanalytic and psychodynamic therapy [24].

Repression and Defence Mechanisms

Freud's idea of repression, pushing uncomfortable thoughts and memories out of conscious awareness, continues to be influential. Modern therapists acknowledge that people sometimes avoid difficult emotions or memories, but rather than repression, these behaviors are seen as avoidance or emotional suppression [25].

Psychosexual Development

Freud's theory of psychosexual development has largely been replaced by more modern theories of child development, such as Erik Erikson's psychosocial stages. However, Freud's general idea that early childhood experiences shape our later behavior is still accepted [26].

The Oedipus Complex

Freud's concept of the Oedipus complex, where a child unconsciously desires the opposite-sex parent and competes with the same-sex parent, has largely been criticized and is not widely accepted in modern psychology. While most experts do not believe that this theory accurately reflects childhood development, modern attachment theory and family systems theory are examples of approaches that build on this idea without relying on the Oedipus complex [27].

The Id, Ego, and Superego

Freud's structural model of the psyche, dividing the mind into the id (instincts), ego (reality), and superego (morality), is still discussed in some forms of psychotherapy. However, modern psychology looks more at how cognitive processes work, including decision-making, self-control, and morality, without strictly using Freud's framework. Cognitive behavioral therapy (CBT) focuses more on how thought patterns influence behavior [28].

Freud's influence on therapy

Freud's approach to therapy, known as psychoanalysis, is still practiced, but it has evolved into a shorter, more focused method called psychodynamic therapy. This approach helps people understand how their past experiences, especially from childhood, affect their current thoughts and behaviors. Modern therapy often combines Freud's ideas with more evidence-based methods such as CBT, which focuses more on solving problems in the present rather than digging into the past [29].

Sigmund Freud never directly discussed phi neurons or psi neurons in his work, as these concepts were not part of his theory of the mind. However, in modern neuroscience and psychoanalysis, some theorists have tried to connect Freud's ideas with newer concepts such as "phi" and "psi" neurons.

Phi Neurons and Freud's Conscious Mind

In modern neuroscience, "phi" often refers to the integration of information in the brain that leads to consciousness (as in integrated information theory) [30].

In Freud's terms, consciousness is where we are aware of our thoughts, feelings, and surroundings. If we link this to Freud, we could imagine phi neurons as representing the brain's activity when we are conscious of our thoughts, the thoughts that are right at the surface, what Freud called the conscious mind [20].

Psi Neurons and Freud's Unconscious Mind

In some theories, psi neurons could represent processes related to the unconscious mind, where mental activities happen outside of our awareness. Freud famously emphasized the importance of the unconscious, which he believed was full of repressed thoughts, desires, and memories that influence our behavior [30]. Psi neurons, in this analogy, might symbolize the brain's activity involved in processing information that we are not consciously aware of, much like Freud's unconscious mind [15]. This could include things such as suppressed emotions or forgotten memories, which Freud said have a big impact on our behavior and mental health [14].

Phi neurons represent conscious processing (Freud's conscious mind), and psi neurons represent unconscious processing (Freud's unconscious mind).

Cathexis (Mental Energy Investment)

In Freud's view, cathexis refers to the idea that we mentally invest energy into people, objects, or ideas [15]. This energy comes from our desires and needs, and it is directed toward things we care about or want. For example, if you love someone, your mind "invests" energy in that person [16]. This can also happen with objects, like when someone becomes obsessed with a hobby or collects things; energy is mentally tied up in those interests.

Freud believed that this energy comes from our drives, such as the libido (the sexual energy that motivates much of our behavior) or aggressive drives. When we focus a lot of mental energy on something, we experience cathexis [16]. However, if this energy becomes too intense or misplaced (e.g., fixating too much on someone), it can lead to problems such as obsession or anxiety.

Cathexis is like an "emotional investment," when you put your mental energy into something you care about.

Catharsis (Emotional Release)

Catharsis is the process of releasing strong emotions that have been suppressed or held inside [6]. Freud believed that sometimes we push painful feelings into our unconscious mind to avoid dealing with them (this is repression) [25]. However, when those emotions stay bottled up for too long, they can cause mental distress.

Freud thought that releasing these hidden emotions through therapy, such as talking about them or reliving them, helps reduce the emotional burden and leads to healing. This emotional release is catharsis [6]. Catharsis is a way to get relief from repressed emotions. For example, when someone cries after talking about a difficult experience, they might feel better afterward.

Catharsis is the emotional "release" or relief you feel when you finally express emotions that you have been holding inside.

How Do Cathexis and Catharsis Work Together?

In Freud's theory, people often cathect (invest energy) into desires, objects, or relationships. If that energy leads to frustration or emotional buildup (e.g., if someone cannot have what they want), it may create tension or anxiety. Catharsis happens when that energy is released in a healthy way, such as through talking about it or expressing emotions, leading to relief [6].

Cathexis: A person deeply in love with someone who does not love them back might cathect a lot of energy into thinking about that person, causing frustration or sadness.

Catharsis: After discussing their feelings in therapy, the person might cry or express their emotions, experiencing a sense of relief.

Impact of Sigmund Freud's father on his work

Sigmund Freud's relationship with his father, Jakob Freud, had a profound influence on his theories. Freud often wrote about the impact his father had on his thoughts and feelings, particularly in shaping his ideas about authority, power, and the role of the father in family life [18]. Freud's relationship with his father was complex; he admired him but also felt ambivalence, which he later connected to his theory of the Oedipus complex [16]. This concept suggests that children may experience feelings of competition with their same-sex parent for the attention of the opposite-sex parent.

Freud's reflections on his father helped him develop this key theory in psychoanalysis. Freud's father also influenced his thinking about death, legacy, and the passing of time. Jakob Freud's death in 1896 deeply affected Sigmund, and this personal loss contributed to Freud's increased focus on mourning, loss, and the unconscious mind's coping mechanisms [31].

Impact of Sigmund Freud's daughter, Anna Freud, on his work

Anna Freud, Sigmund Freud's youngest daughter, not only supported her father's work but also extended and refined many of his ideas. Anna became a prominent psychoanalyst in her own right and focused much of her work on child psychology and defense mechanisms [32].

While Sigmund Freud laid the foundation for defense mechanisms, such as repression, Anna expanded on these concepts and added new ones, such as denial, projection, and displacement.

Anna Freud's work on child development and the ways children cope with anxiety, fear, and stress helped further develop psychoanalytic theory, especially in understanding how children's minds work differently from adults [33].

Her close collaboration with her father toward the end of his life also ensured that Sigmund Freud's theories continued to evolve, particularly in the areas of child psychoanalysis and therapeutic techniques for children [34].

Sigmund Freud's theories have had a huge impact on psychology, but they have also faced significant criticism over the years. Many of his ideas have been criticized or replaced by more modern approaches. His focus on sexuality, the unconscious mind, and childhood has been questioned. Despite these critiques, Freud's work remains influential, and his contributions continue to shape the field of psychology [35].

Karen Horney challenged Freud's views on female psychology, particularly his concept of "penis envy." Horney argued that Freud's theories were rooted in a male-centric perspective and did not accurately reflect women's experiences or psychological development. She believed that women's feelings of inferiority were more about social and cultural factors rather than biological envy [36].

Erik Erikson, known for his theory of psychosocial development, criticized Freud's focus on psychosexual stages. Erikson argued that Freud placed too much emphasis on early childhood and sexuality, and he proposed that personality development continues throughout life, with social and cultural factors playing a significant role [37].

Carl Rogers, a leading figure in humanistic psychology, criticized Freud's approach for being too deterministic and focused on pathology. Rogers advocated for a more positive view of human nature, emphasizing self-actualization and the potential for growth rather than focusing on unconscious conflicts [38].

B.F. Skinner, a prominent behaviorist, criticized Freud for his lack of empirical evidence and scientific rigor. Skinner believed that behavior could be understood through observable actions and conditioning, rather than exploring unconscious motives and internal conflicts [39].

Anna Freud, Sigmund Freud's daughter, was a prominent psychoanalyst in her own right. Although she supported many of her father's ideas, she also offered critiques and modifications to his theories. She largely accepted her father's concepts of defense mechanisms but elaborated on the mechanisms and explored their role in coping with anxiety and maintaining psychological balance [32].

Sigmund Freud's theories, particularly his ideas on the unconscious mind, defense mechanisms, and psychosexual development, laid the groundwork for many other psychological theories [4]. Freud's pioneering concepts influenced not only psychoanalysis but also the development of other key psychological fields such as behaviorism, cognitive psychology, and humanistic psychology. For instance, Carl Jung's theory of archetypes, Erik Erikson's psychosocial development, and even cognitive behavioral therapy (CBT) emerged in part as responses to or evolutions from Freud's original work [40]. While Freud's ideas have been criticized and refined over time, they sparked critical thought that shaped modern psychology's understanding of human behavior, mental processes, and emotional development [32].

**Figure 1:**
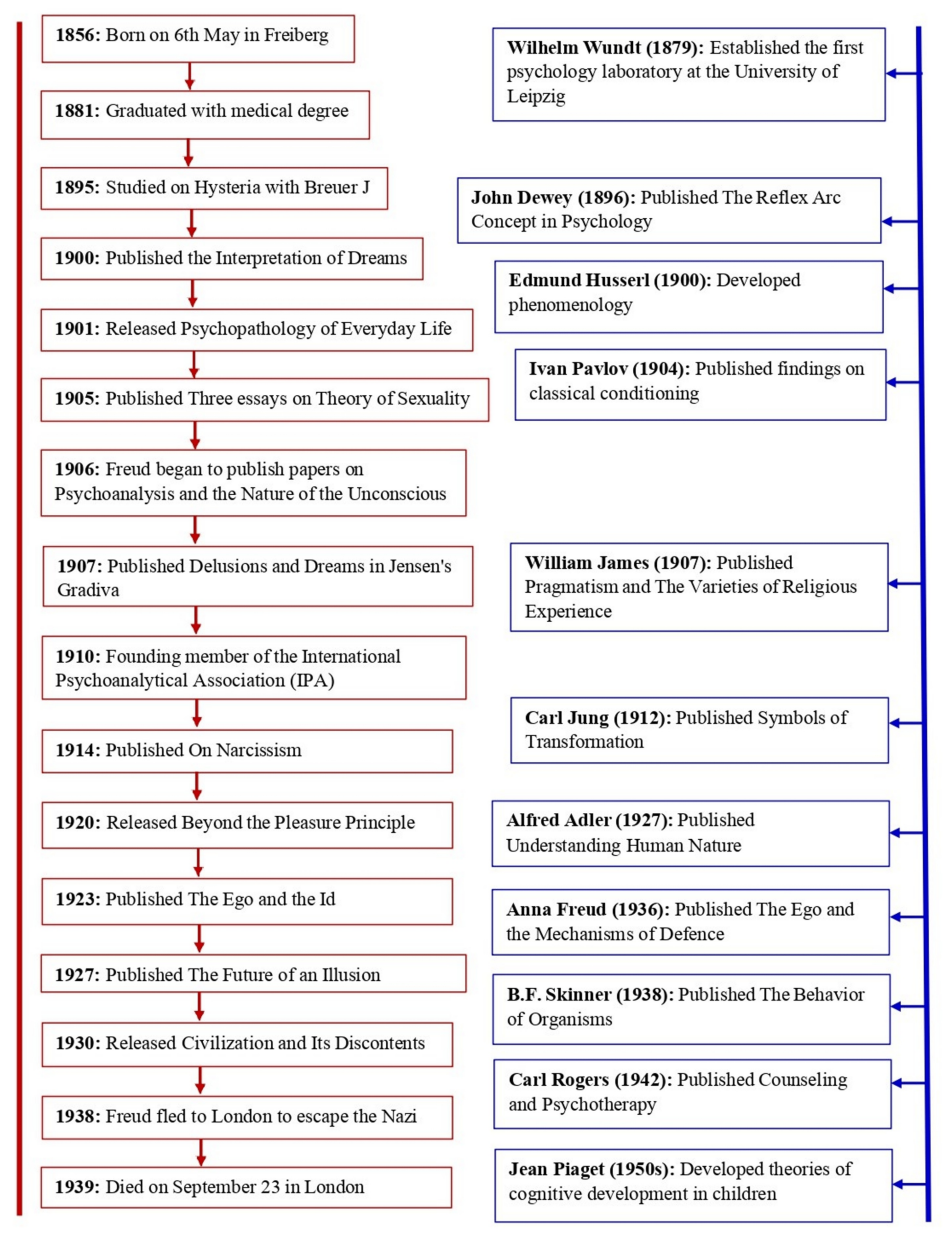
Timeline showing life and achievements of Sigmund Freud along with parallel scientific advances in psychology

A timeline of Sigmund Freud's life and major achievements highlights his contributions to psychoanalysis, alongside key parallel advances in psychology. It traces his work from the development of foundational theories to the publication of The Interpretation of Dreams, while also noting contemporary scientific milestones that shaped the broader field (Figure 1).

## Conclusions

In conclusion, Sigmund Freud's life and contributions have had a profound impact on psychology and beyond. His groundbreaking theories have had a significant impact on our comprehension of human behavior and mental processes. These theories include the investigation of the unconscious mind, the formation of psychoanalysis, and the clarification of complicated psychological phenomena. Freud's journey from his early days in Vienna to becoming a leading figure in the field of psychology was defined by a persistent pursuit of knowledge and an unshakeable commitment to exploring the nuances of the human psyche. Although he encountered strong resistance and debate, his inventions revolutionized our understanding of human development and mental health.

The applicability of Freud's theories today, the continuous discussions they spark, and the fundamental part they play in a range of therapeutic approaches are all clear indications of his legacy. His work has opened doors for a deeper investigation of the hidden aspects of the mind for upcoming generations of psychologists, researchers, and doctors.
